# Knowledge and practice on oral hygiene maintenance among medical students in India

**DOI:** 10.6026/973206300211226

**Published:** 2025-05-31

**Authors:** Bharat Jayant Sumbh, Shweta Gangotri, Rishi Thukral, Heeralal Chokotiya, Ashtha Arya, Ajay Kumar

**Affiliations:** 1 Departmentof Dentistry, AtalBihari Vajpayee Government Medical College and Hospital, Vidisha, Madhya Pradesh, India; 2 Departmentoral Medicine & Radiology, Government Dental College, Nagpur, Maharashtra, India; 3 Department of Conservative Dentistry and Endodontics, SGT Dental College, Hospital and Research Institute, SGT University, Gurugram, Haryana- 122505; 4 Department of Periodontology, Eklavya Dental College and Hospital, Jaipur, Rajasthan, India

**Keywords:** Awareness, dental health, medical students, oral hygiene

## Abstract

Oral hygiene is vital in preventing dental caries, periodontal diseases and systemic conditions like stroke and heart disease. Therefore, it is of interest to evaluate oral health awareness among MBBS students and interns using a pretested, structured questionnaire. Data analysis was performed using the Chi-square test with a significance threshold of 0.05. A significant gender difference was observed in brushing frequency (P = 0.005) and only 36.9% of students reported brushing twice daily. Hence, there is a need to enhance oral health education in medical curricula for effective preventive healthcare.

## Background:

Oral health is an important aspect of general health and social well-being [1]. It affects an individual's oral functions and is closely linked to overall health and quality of life [2, 3-4]. The oral cavity is a well-known nidus of infection leading to dental caries and periodontal problems due to poor oral hygiene practices. Thus, oral hygiene, if adopted properly, can help get rid of the majority of oral diseases [5, 6]. The oral and systemic health is closely associated with each other, so the systemic diseases will be exacerbated if oral disease is left untreated [7]. Proper awareness, knowledge and motivation regards oral health practices leads to excellent oral hygiene [8, 9]. Medical practitioners being the primary healthcare providers in the community could significantly impact the patients' oral health by providing awareness regarding oral health. They can act as role models for their family and for the community at large by integrating oral health awareness in their practices [10, 11]. Assessment of oral health awareness amongst medical students is essential because the undergraduate students need to know the importance of oral health and the conglomeration that exists between oral and general health [12]. Therefore, it is of interest to assess the oral health awareness amongst medical students.

## Methods:

The present cross-sectional survey study was conducted amongst 225 MBBS Students and Interns from April 2023 to May 2023 at Government Medical College. As the study was an observational study (questionnaire survey) and no intervention was done, therefore ethical clearance is not required as per the ethical guidelines of the Declaration of Helsinki and the Indian Council of Medical Research. All the students who were willing to participate were included in the study. Those who were not willing to participate were excluded from study. After taking their informed consent, a total of 225 students present on the day of data collection were included in it. Confidentiality regarding the responses was maintained throughout the study. The study tools do predesigned, pretested and structured self-administered questionnaires comprise 12 questions which were used to measure the oral health awareness of the medical students in this study. The self-administered questionnaire has been converted into Google Forms to collect data online. The Google form link had been sent to the study participants who were MBBS students and Interns of Government Medical College through WhatsApp. The participants were requested not to discuss amongst their friends while marking the responses, as our objectives were to know the individual responses. Data was recorded in MS Excel and checked for its completeness and correctness, then it was analyzed using suitable statistical software and the p value <0.05 was considered as statistically significant. The Chi-square test was used to analyze the data and the level of significance was at 0.05 and the sample size was calculated according to the above formula.

Sample size estimation formula:-

n = Z^2^_1_x P (1-P) / d^2^

## Results:

In this present study, 225 participants responded to the questionnaire. The proportions of demographic data for the Male: Female ratios were 56.5:43.5 (Table 1). The native place of students from urban areas was 68.2%, and from rural areas was 31.8% (Table 2). The students had a mean age of 22 years. Good oral hygiene was reported by 64.4% of students, 8.9% of students reported excellent oral hygiene, 24.4% stated their oral hygiene was average, and 2.2% considered it poor. Students cleaning their teeth with brush and paste were 88.9%, while 8.4% used Patanjali tooth powder, and 7.8% used neem, Lal Dant Manjan, finger, Dant Kanti, and Sensoform toothpaste. The frequency of brushing teeth was once daily for 74.8%, twice daily for 23.5% and after every meal for 1.7%. Out of 225 students, 50.5% used soft brushes, 44.4% used medium-quality brushes, approximately 1% used hard brushes, and 4.2% were unsure about their brush type. The frequency of changing toothbrushes was once in three months for 55.1%, once in six months for 27.1%, when it became useless for 13.1%, and once a year for 4.7%. Regarding the method of teeth cleaning, 9.3% used circular motion, 13.1% used horizontal motion, 2.4% used vertical strokes, and 75.2% used a combination of all motions. Among them, 65% of students reported not using any interdental cleaning aids, while 12.1% used toothpicks, 10.3% used floss, and 12.6% used interdental brushes. The majority of students (81.3%) reported not using mouthwash, whereas 13.7% used it. Tongue cleaning was practiced daily by 65% of students, occasionally by 24.3%, never by 9.8%, and only when there was a bad odor by 0.9%. The dental check-up history revealed that 43.5% had never had a dental check-up, 17.8% had it once a year, 21% had visited the dentist once, and 4.1% had a regular dental check-up.

## Discussion:

Dental health is an important issue as bright and healthy teeth are the mirror of a healthy body. Various studies have been conducted to assess the knowledge, attitude and practices of oral hygiene in students with a background of health sciences. Oral health is usually ignored not only by ordinary people but also by medical professionals, so basically, this study was conducted keeping in mind the fact that today's students are tomorrow's doctors [6, 13]. A cross-sectional survey conducted amongst international medical and dental students by Petrauskiene *et al.* depicted an overall good, fair and poor oral health awareness reported by 71.3%, 24.1% and 4.6%, respectively [8]. The findings of the above study are similar to the results obtained in the present study. The present study results showed that only 64.4 % of study participants had good oral hygiene status. The difference between males and females regarding the frequency of tooth brushing was statistically significant (P = 0.005). In the present study, only 36.9% (Table 3) of the medical students reported brushing their teeth twice daily and that was in contrast to the results of Peltzer and Pengpid, who reported it to be 67.2% in university students, 26 low, middle and high income countries [14]. The present observation may be due to the occupancy of the students in their curricular activities and ignorant attitude toward oral hygiene, considering it as less important. In the present study, females brush their teeth more frequently than males. This is in accordance with the studies of El-Qaderi and Taani [15]. It is noteworthy that 76.4 % (Table 3) of the students' tooth brushing method using combined Motion, this finding is in contrary with that of the study done by Zhu *et al.* [16]. Where 60% of the sample used horizontal motion. Only 51.1% (Table 3) of the students use used a soft brush, which is more than that observed amongst Zhu *et al.*'s subjects where 27% of the sample used the same [16].

In the current study, 56.4% of participants reported changing their toothbrush every three months, 25.8% replaced it once every six months, and 4.4% replaced it once a year. These rates are lower than those reported by Sood *et al.* [17]. In their study, Sood *et al.* found that 61% of participants changed their toothbrush after three months of use. However, the percentage of participants who changed their toothbrush after six months in our study (25.8%) was much higher than that reported by Sood *et al.* where only 15.67% of participants did so. This infers that these students are unaware of the fact that prolonged usage of toothbrushes not only decreases effectiveness in cleaning of plaque but also causes trauma to gingival tissue. They should be educated about the importance of changing toothbrushes at regular intervals. Hamilton and Coulby found that a high percentage (44%) studied in North Eastern Ontarious dental floss. In contrast, this current study reported only 9.8 % (Table 3) of students using dental floss [18]. Dentists play a major role in maintaining overall dental health. Nearly 20.4% of the medical students had at least visited their dentist once in <6 months, which was nearly consistent with the result of studies by Doshi *et al.* and Al-Hussaini *et al.* [19, 20]. Almost 43.6% of individuals had never been to a dentist, which was in consonance with the study done by Gopikrishna *et al.* amongst engineering students of Bangalore [21]. A still lower percentage of students 17.8% had visited a dentist within 1 year. The study by Shanmugham *et al.* (2024) investigated the knowledge and practice of oral hygiene maintenance among dental students. It highlighted that while dental students possess substantial theoretical knowledge about oral hygiene, there remains a gap in consistently applying these practices in their personal routines. The research emphasizes the need for improved educational interventions that not only focus on theoretical aspects but also encourage the practical application of oral hygiene measures. This could lead to better personal oral health habits among future dental professionals, ultimately enhancing patient care standards [22].

## Limitation of the present study:

This study was an online questionnaire survey. There were certain limitations, like a poor response rate, difficulty in judging the seriousness of the responses. The sample size in the current study was 225 medical students. To generalize the findings of the current study, the study should have been conducted by a large number of medical students amongst different medical institutes for greater validity of current findings. Other than medical students, the paramedical and nursing students could also be considered for the study.

## Future prospects:

Awareness and continuing medical education trainings regarding oral health and diseases should be implemented in the educational curriculum of the respective departments.

## Conclusion:

The need to incorporate oral health education into the MBBS curriculum to improve students' awareness is shown. Regular exposure to dental health programs and continuing education will further enhance their ability to address oral health issues. Ultimately, better oral health knowledge among medical professionals will lead to improved patient care and early intervention.

## Figures and Tables

**Table 1:** Demographic characteristics of the study subjects

**Characteristics**		**n**	**%**
sex	Male	127	56.50%
	Female	98	43.50%
Total		225	100%

**Table 2:** Native regions of the study subjects

**Characteristics**		**n**	**%**
	Urban	153	68.20%
			
	Rural	72	31.80%
Total		225	100%

**Table 3:** Association of gender with other attributes

**SN**	**Attributes**		**Gender**		**Total (%)**	**Total (n)**	**Chi-square, df, p value**
			**Female**	**Male**			
I	Oral hygiene status	Average	27.00%	22.40%	24.40%	55	1.752, 30.626
		Excellent	7.00%	10.40%	8.90%	20	
		Good	63.00%	65.60%	64.40%	145	
		Poor	3.00%	1.60%	2.20%	5	
II	Teeth cleaning	Brush and paste	93.00%	85.60%	88.90%	200	6.866, 50.231
		Chewing neem stick	0.00%	1.60%	0.90%	2	
		Dantkanti	0.00%	0.80%	0.40%	1	
		Laldantmanjan	0.00%	1.60%	0.90%	2	
		Patanjali toothpowder/paste	6.00%	10.40%	8.40%	19	
		Sensoform	1.00%	0.00%	0.40%	1	
III	Frequency of tooth brushing	After every meal	2.00%	0.80%	1.30%	3	10.666, 20.005
		once	50.00%	71.20%	61.80%	139	
		twice	48.00%	28.00%	36.90%	83	
IV	Type of tooth brush	Don't know	2.00%	6.40%	4.40%	10	5.546, 30.136
		Hard	0.00%	0.80%	0.40%	1	
		Medium	40.00%	47.20%	44.00%	99	
		Soft	58.00%	45.60%	51.10%	115	
V	Changing tooth brush	once in 3months	52.00%	60.00%	56.40%	127	5.813, 30.121
		once in 6 months	32.00%	20.80%	25.80%	58	
		Once in a year	2.00%	6.40%	4.40%	10	
VI	Tooth brushing method	Circular	12.00%	6.40%	8.90%	20	4.171, 30.244
		Combined	76.00%	76.80%	76.40%	172	
		Horizontal	9.00%	15.20%	12.40%	28	
		Vertical	3.00%	1.60%	2.20%	5	
VII	Inter dental aid	Floss	14.00%	6.40%	9.80%	22	7.626, 30.054
		inter dental brush	7.00%	16.80%	12.40%	28	
		None	66.00%	64.80%	65.30%	147	
		Toothpick	13.00%	13.00%	12.40%	28	
VIII	Use of mouth wash	No	79.00%	75.20%	76.90%	173	0.451, 10.502
		yes	21.00%	24.80%	23.10%	52	
IX	Tongue cleaning	Everyday	75.00%	58.40%	65.80%	148	7.195, 30.066
		Never	6.00%	12.00%	9.30%	21	
		Occasionally	18.00%	28.80%	24.00%	54	
		When your mouth smells	1.00%	0.80%	0.90%	2	
X	Dental visit	Any other	17.00%	10.40%	13.30%	30	6.744, 40.15
		Never	35.00%	50.40%	43.60%	98	
		Once per 6 month	24.00%	17.60%	20.40%	46	
		Once per year	20.00%	16.00%	17.80%	40	
